# The Effects of an Individual-Level Strategy on Adoption and Completion of Trauma-Focused Cognitive Behavioral Therapy in Schools: Results from a Hybrid Type 2 Randomized Trial

**DOI:** 10.21203/rs.3.rs-11001359/v1

**Published:** 2026-10-13

**Authors:** Kathryn E. Bruzios, Maria L. Hugh, Mahima Joshi, Larissa M. Gaias, Michael Pullmann, Rosemary Reyes, Ana Paula Martinez Cueto, Roger Goosey, Yasmín Landa, Catherine M. Corbin, Rachel Barrett, James L. Merle, Madeline Larson, Clayton R. Cook, Aaron R. Lyon

## Abstract

Background School-based mental health (SBMH) providers face challenges adopting high-quality evidence-based practices (EBPs) like Trauma-Focused Cognitive Behavioral Therapy (TF-CBT). Individual-level factors (e.g., attitudes) are key implementation determinants, but little is known about how strategies targeting these mechanisms affect not only whether, but when, providers implement EBPs. *Beliefs and Attitudes for Successful Implementation in Schools* (BASIS) is a theory-driven, evidence-based strategy targeting individual-level mechanisms (e.g., attitudes, self-efficacy). This study compared BASIS to an attention control (AC) condition on SBMH providers’ implementation mechanisms, TF-CBT adoption and completion, and their pace. Methods In a hybrid type 2 effectiveness-implementation randomized trial, 92 SBMH providers received BASIS or AC alongside TF-CBT training and consultation. Implementation mechanisms and outcomes (screener adoption, TF-CBT adoption, and TF-CBT completion with at least one student) were assessed at 13 timepoints across pre-implementation (T0-T5), active implementation (T6-T9), and sustainment (T10-T12). Mechanisms were analyzed using mixed-effects models; adoption/completion outcomes and pacing were evaluated using time-to-event analyses, chi-square tests, and Mann-Whitney U tests. We also explored whether mechanisms predicted pacing using a non-parametric GLM gamma log link. Results BASIS had minimal impact on hypothesized mechanisms but significant effects on some adoption pacing. BASIS providers had higher injunctive ( *p* =.015) and descriptive ( *p* =.013) norms than AC overall, though norms did not differ by phase (all *p* -values>.05). Conditions did not differ in attitudes or self-efficacy over time (all *p* -values>.05). Implementation intentions fluctuated, decreasing during active implementation ( *p* <.001) and increasing in sustainment ( *p* =.033). By the end of active implementation, BASIS providers were more likely to have administered a screener (85% vs. 62.5%; *p* =.022) and marginally more likely to have adopted TF-CBT (80% vs. 60%; *p* =.051) but did not differ from AC in TF-CBT completion (27.5% vs. 30%; *p* =.805). Contrary to hypotheses, providers with more positive attitudes and self-efficacy post-TF-CBT training took longer to administer their first screener, regardless of condition. Conclusions Schools increase youth access to mental health services, but EBPs must be implemented quickly to fit a school year’s time constraints. BASIS accelerated screener adoption, but further research is needed on its potential to influence TF-CBT adoption and completion pacing. Implementation mechanisms may shift across the implementation timeline, and strategies are needed to align with key implementation windows. Trial registration ClinicalTrials.gov ID NCT04451161. Registered on 6/26/2020.

**Authors' contributions:** KEB, MLH, ARL conceptualized the manuscript. KEB and MLH wrote the main manuscript text with support from ARL, MJ, and LMG. MJ, LMG, MP curated and conducted the formal analyses. RB, MLH, CMC, JLM, and ML conducted the research process and RG, MJ, YL, and RR collected data and project administration. APMC, MJ, KEB, and MLH created all tables and figures. ARL and CRC acquired funding for this study. All authors reviewed the manuscript.

As youth mental health needs grow in complexity, schools, the primary setting for service delivery, are increasingly positioned as "one-stop-shops" for comprehensive supports.[1,2] Unfortunately, evidence-based practices (EBPs) that address these needs remain insufficiently implemented in under-resourced schools.[3,4] Moreover, the school-based mental health (SBMH) providers (e.g., counselors, social workers) who often deliver these EBPs have growing responsibilities, burdens, and barriers to use.[4–7] Importantly, providers' knowledge, skills, and beliefs, ultimately influence whether EBPs are delivered.[8–10] Yet, even as our understanding of beliefs as implementation drivers grows, less attention has been given to *when* implementation happens; particularly through dynamic mechanisms like beliefs to ensure timely access, successful implementation, and improved youth outcomes.

**Individual-Level Determinants to School-based Implementation**

Even in supportive organizations, individual-level factors are critical determinants of implementation success.[5,11,12] Despite gold-standard EBP training and high provider knowledge and skill in new practice, providers' beliefs can impede implementation.[8] Over time, these barriers contribute to delayed adoption or incomplete implementation, reducing EBP impact and straining limited resources. Encouragingly, a systematic review found that school-based providers' engagement and commitment to delivery were key facilitators of EBP implementation.[13] Because individual-level factors may be more malleable than organizational-level factors,[8,14,15] targeting them is promising for improving uptake and potentially the pace at which SBMH providers adopt and complete implementation.[16]

The facilitating and inhibiting effects of individual attitudes and beliefs are grounded in a the Theory of Planned Behavior (TPB).[17] The TPB illustrates that providers' attitudes (i.e., favorable or unfavorable evaluation of a behavior), subjective norms (i.e., perceived social expectations for the behavior), and perceived behavioral control/self-efficacy (i.e., perceived ease or difficulty to do the behavior) shape their intention to implement a new practice, with stronger intentions more often leading to implementation. Although the TPB describes the sequential relationship between beliefs, intention, and implementation, real-world implementation is an ongoing, multi-stage, and reciprocal process wherein beliefs may, and often should, change over time.

**Improving the Pace of Implementation in Schools**

The persistent gap between learning about, adopting, and fully implementing EBPs[18–20] requires attending to the *pace* of implementation in critical service delivery contexts at both individual- and organizational-levels. Implementation pace represents the speed of accomplishing milestones[18] within and across phases of implementation.[7] Pacing should balance speed and efficiency with process steps, considering contextual factors, EBP complexity, and competing priorities.[21] Yet, pace is rarely represented in implementation outcome taxonomies.[22] Though recent literature addresses rapid implementation[23] and frameworks exist to design and assess speed (e.g., the Framework to Assess the Speed of Translation),[18,24] their application is limited, particularly in educational contexts. Understanding pace is particularly important when scaling out mental health EBPs to settings and implementers that differ from the original clinical research context, where contextual and individual factors may introduce additional delays.[25] In kindergarten through twelfth (K-12) grade schools in the

United States (US), the timing gap becomes more pronounced due to structural time constraints that distinguish schools from other youth mental health service delivery settings.

For individual implementers, the fixed start time of the academic year presents unique demands that may influence EBP adoption and later completion. For example, a frequent challenge in school-based implementation is starting or initiating an EBP for the first time and overcoming obstacles to implementation over time.[26] In the beginning of the school year, providers often face compounding organizational barriers of new initiatives, caseloads, schedules, and colleagues;[27] all of which demand critical and time-consuming activities for EBP delivery including difficult-to-access information, screening and identification for mental health supports, consultation, and relationship-building.[28] In turn, these activities require *motivation* to initiate these behaviors and persist through each step of EBP implementation.[16,26] Taken together, the interaction of the organizational context, demands, and EBP factors constrain an individual implementers' opportunities to implement the EBP, and their beliefs and motivation are required to buffer and overcome these constraints, initially and over the course of the school year. Although implementation strategies exist targeting individuals' adoption and implementation,[29] few target the mechanistic individual drivers (i.e., beliefs) or help plan for the necessary pacing of implementation. Therefore, strategies targeting SBMH providers' beliefs and motivation are needed to accelerate adoption and completion of EBPs.[8,26,30,31]

**Beliefs and Attitudes for Successful Implementation in Schools (BASIS)**

*Beliefs and Attitudes for Successful Implementation in Schools* (BASIS) is a theory-driven implementation strategy targeting individual-level determinants to improve implementation.[14,26,30,31] BASIS (Figure 1) draws on the Theory of Planned Behavior,[17]

the Health Action Process Approach,[16] and research that providers' beliefs and implementation can change based on other malleable factors such as working conditions, student characteristics, having envisioned and planned for implementation steps (i.e., action planning), past behavior or implementation, and pedagogical practice.[32–34] Within group-based sessions immediately before and after EBP training, BASIS incorporates motivational strategies including strategic education, social influence, Motivational Interviewing (MI), action planning, and problem-solving planning, to enhance these mechanisms and thereby strengthen implementation intentions and outcomes (see Hugh et al., 2026 for details of the motivational strategies).[17,30,35,36]

Prior BASIS studies demonstrate meaningful impact on hypothesized behavior change mechanisms (self-efficacy, attitudes), both immediately after BASIS and over time, for universal prevention EBPs;[26,30,31] these mechanisms have been associated with improved provider adoption and fidelity across different interventions.[26,30,31,35] Prior to this trial, BASIS was facilitated in-person by expert researchers with practitioner experience, within the school-wide implementation of a universal positive behavior support program.[31] These trials demonstrate that BASIS providers had significantly higher self-efficacy and adoption of a classroom-wide positive behavior support intervention relative to an attention control condition at one-month follow-up, with promising impacts on student behavioral outcomes.[26] The efficacy of BASIS as an 'EBP-agnostic' strategy (i.e., one that can be applied across a variety of EBPs) motivated us to evaluate its effectiveness when delivered in a more feasible, school-based manner. This included high-fidelity BASIS delivery: a) by a non-researcher (practitioner) facilitator, b) via web-based Zoom sessions, and c) over a shorter duration, while maintaining core components: Motivational Interviewing techniques to elicit, reflect on, and affirm a need for change, cognitive dissonance, and “change talk”; goal-setting; and action- and coping-planning (5-10 hours; see Hugh et al., 2026 for detailed delivery description). Given that school-based providers may have variable and diminishing implementation over time, BASIS may be a particularly important tool for building supportive beliefs and helping providers overcome barriers as they arise.[26,30,31,35]

Figure 1

*Beliefs and Attitudes for Successful Implementation in Schools (BASIS) Theory of Change*

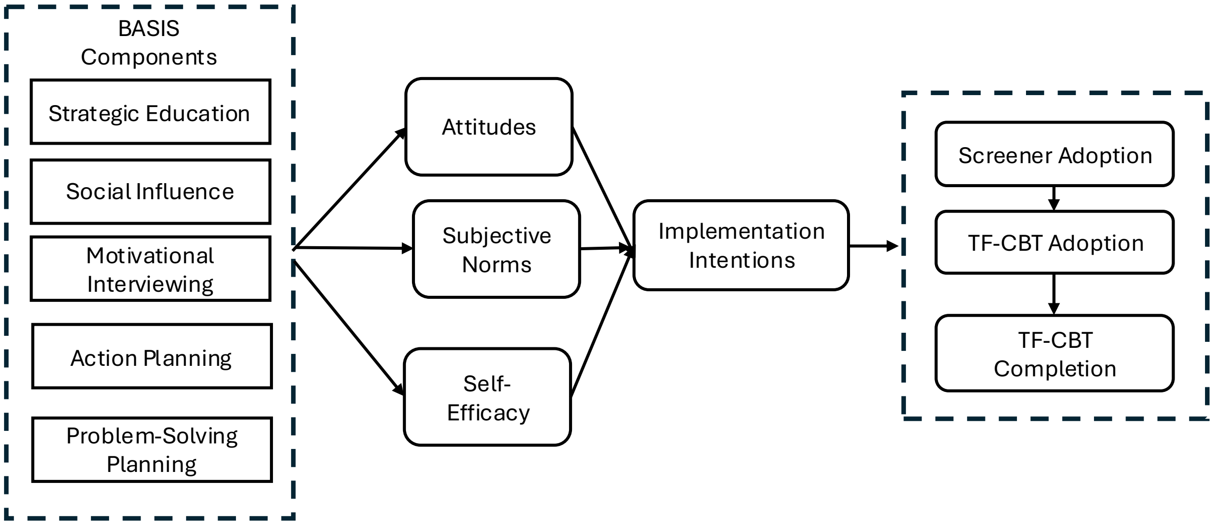

*Note.* BASIS = Beliefs and Attitudes for Successful Implementation in Schools; TF-CBT = Trauma-focused Cognitive Behavioral Therapy

**Present Study**

Prior BASIS pilot studies offer insights into its potential to accelerate the timing of providers’ implementation steps, persistence, and continued EBP delivery.[26,30,31] Evaluating whether, how, and when BASIS can accelerate implementation within constrained school years can inform understanding of implementation pace. Through a hybrid type 2 effectiveness-implementation randomized controlled trial,[37] we evaluated whether BASIS improves SBMH providers’ adoption and completion of Trauma-Focused Cognitive Behavior Therapy (TF-CBT). TF-CBT is a well-established yet insufficiently implemented EBP for youth with clinicallysignificant symptoms of post-traumatic stress,[12,38–40] with individual-level beliefs as demonstrated barriers to SBMH providers' use.[41] This paper reports BASIS effects on: 1) theorized proximal implementation mechanisms (attitudes, subjective norms, self-efficacy, intentions), and 2) adoption and completion outcomes and pacing among SBMH providers adopting and completing TF-CBT (Figure 1). Specifically, this study addressed three research questions:

1) *Relative to the attention control (AC) condition, did BASIS providers experience more positive implementation mechanisms (attitudes, norms, self-efficacy, intentions)? Did this effect vary by timepoint?*

2) *Relative to AC, were BASIS providers more likely to adopt and complete TF-CBT including screener adoption, TF-CBT adoption, and TF-CBT completion over the study period? Did this effect vary by timepoint?*

3) *Across conditions, how did implementation mechanisms affect the pacing of adoption and completion outcomes (time to screener adoption, time to TF-CBT adoption, time to TF-CBT completion)?*

### Methods

We conducted a hybrid type 2 effectiveness-implementation randomized trial[37] to evaluate the impact of BASIS, relative to an attention control (AC) condition, on SBMH providers' adoption and completion of TF-CBT for trauma-exposed youth. Design, procedure, and measurement details are reported in our protocol.[36]

#### Recruitment and Randomization

We recruited 205 SBMH providers via district administrators across three years (Figure 2 for CONSORT Diagram). Providers were eligible if they 1) held a master's degree or equivalent certification and 2) had not previously participated in TF-CBT or closely related training (i.e., CBT+[42]). Of those interested, 127 providers consented and enrolled across 112 unique schools in 23 districts. Enrollment by Year was: Year 1 (2020-2021), 28 schools, 28 providers; Year 2 (2021-2022) 45 schools, 46 providers[1]; and Year 3 (2022-2023), 39 schools, 53 providers. This study includes the participants from the two TF-CBT randomization conditions; a third condition in the larger study involved a different clinical intervention (see Lyon et al., 2021 for details of the full trial).

During Years 1 and 2, one provider per school was recruited and randomized at the individual-level, with stratification on both school- and provider-level characteristics including baseline implementation mechanisms (i.e., attitudes, norms, self-efficacy, intentions), SBMH provider years of experience, and school characteristics (i.e., size, attendance, free/reduced lunch rates, percentage White, disciplinary rates). In Year 3, up to three providers per school were recruited and randomized using the same school- and provider-level characteristics, with providers within the same school assigned to different conditions. Stratified randomization and a nearest neighbor algorithm created paired distance estimates between consented providers using baseline implementation mechanisms, SBMH provider years of experience, and school characteristics. Matched groups were then created based on smallest distance estimates, resulting in 1-3 BASIS and 1-3 AC groups per year (*n* = 2-10 providers per group), exceeding our minimum sample size of 70 assuming power of .80. To minimize cross-condition contamination among providers particularly those who were co-located within the same school building, providers and TF-CBT consultants were blinded to condition assignment. Moreover, TF-CBT

[1] In Year 2, two providers enrolled in the study from the same school who were both randomized to the same condition due to the research team not having extended the criteria until Year 3. These providers served different grade levels within the school as it was a small school district.

consultation groups were formed within condition, such that providers received consultation alongside others who were assigned to the same condition. Of the 127 enrolled providers, 92 completed TF-CBT training, with 47 receiving BASIS and 45 receiving AC. Thirty-three providers across both conditions were lost to follow-up (Figure 2).

Figure 2

*CONSORT Flow Diagram of Provider Enrollment, Allocation, Follow-up and Analysis*

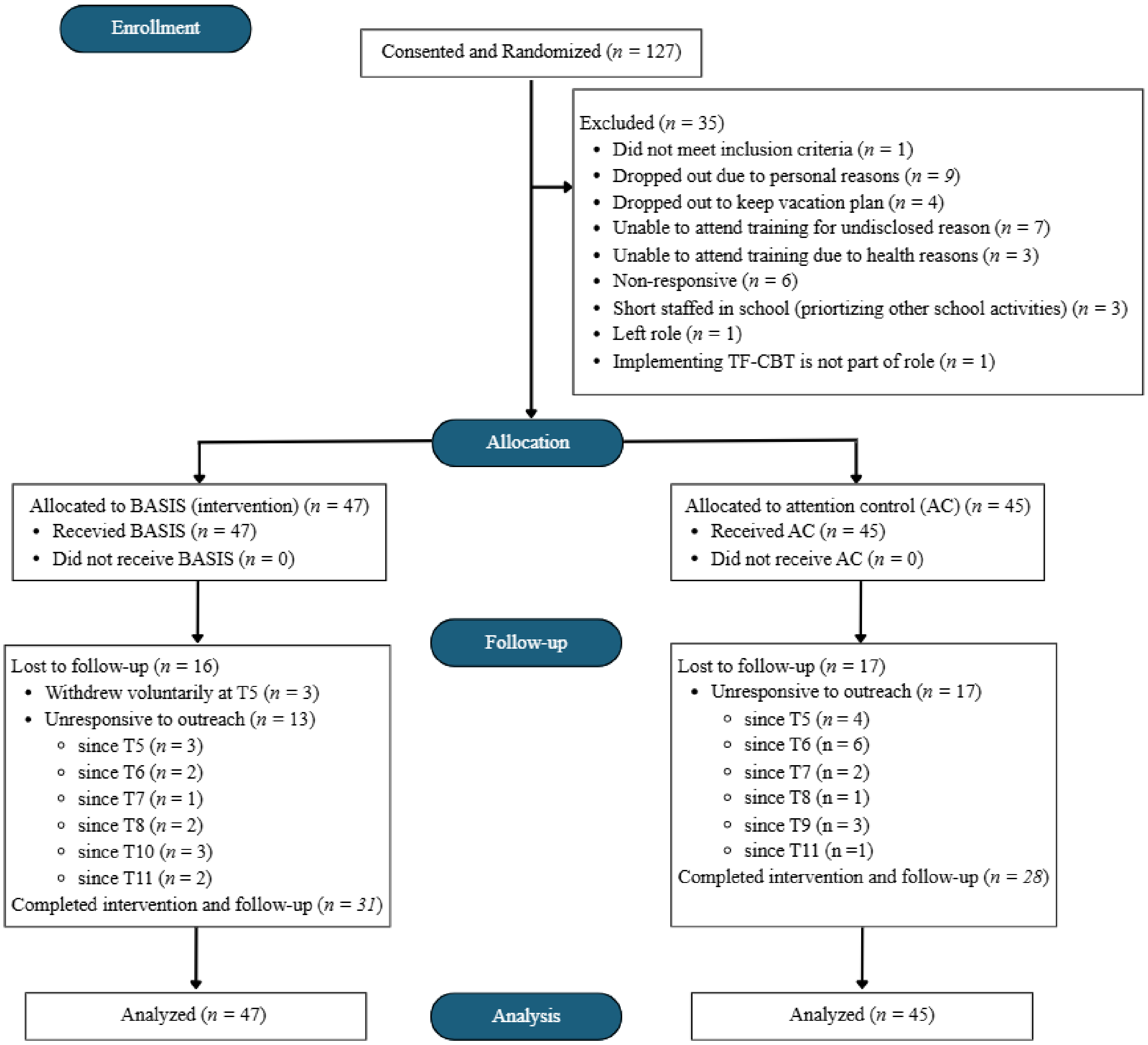

### School-Based Mental Health Providers

SBMH providers included social workers, counselors, and psychologists (*n* = 127) from 23 districts across eight states representing all US regions. School levels included elementary (59.57%), middle (12.77%), high (14.18%), combined (2.13%), and alternative (11.35%; i.e., virtual academies, central offices, etc. that support students from elementary school to young adulthood). Schools varied in enrollment (*M* = 644.08, *SD* = 410.18, Max = 2,049, Min = 124), average student attendance rate (*M* = 86.74%, *SD* = 13.64, Max = 98.80%, Min = 24.46%), free/reduced lunch eligibility[43] (*M* = 36.6%, *SD* = 23.18, Max = 86.21%, Min = 0%), English language learner population (*M* = 41.2%, *SD* = 40.61, Max = 98.5%, Min = 0%), and racial and ethnic composition (*M* = 57.0% White, *SD* = 21.75, Max = 82%, Min = 10%).

SBMH provider demographics for the full sample and by condition are shown in Table 1. Most providers were female (92.4%), White/Caucasian (87.0%), and non-Hispanic/Latina/o/x (96.7%), and held a master's degree or higher (72.8% master's only). Condition differences were non-significant on all demographic variables (Table 1).

Table 1

*SBMH Provider Characteristics and BASIS Mechanisms Descriptive Statistics*

| | **BASIS Providers (*n* = 47) *N* (%)** | **AC Providers (*n* = 45) *N* (%)** | **Total (*n* = 92) *N* (%)** | ***p*-value[a]** |
|---|---|---|---|---|
| Age | | | | .386 |
| 18 to 24 | 0 (0.00) | 1 (2.22) | 1 (1.09) | |
| 25 to 34 | 13 (27.66) | 12 (26.67) | 25 (27.17) | |
| 35 to 44 | 14 (29.98) | 17 (37.78) | 31 (33.7) | |
| 45 to 54 | 17 (36.17) | 9 (20.00) | 26 (28.26) | |
| 55 to 64 | 3 (6.38) | 5 (11.11) | 8 (8.70) | |
| 65 to 74 | 0 (0.00) | 1 (2.22) | 1 (1.09) | |
| Gender | | | | .256 |
| Female | 45 (95.74) | 40 (88.89) | 85 (92.39) | |
| Male | 1 (2.13) | 4 (8.89) | 5 (5.43) | |
| Non-binary/third gender | 1 (2.13) | 0 (0.00) | 1 (1.09) | |
| Unknown or not reported | 0 (0.00) | 1 (2.22) | 1 (1.09) | |
| Race | | | | .827 |
| Asian | 1 (2.13) | 1 (2.22) | 2 (2.17) | |

| | BASIS Providers ($n = 47$) N (%) | AC Providers ($n = 45$) N (%) | Total ($n = 92$) N (%) | p-value[a] |
|---|---|---|---|---|
| Black or African American | 1 (2.13) | 2 (4.44) | 3 (3.26) | |
| Native Hawaiian or Pacific Islander | 0 (0.00) | 1 (2.22) | 1 (1.09) | |
| White or Caucasian | 42 (89.36) | 38 (84.44) | 80 (86.96) | |
| More than one race | 2 (4.26) | 1 (2.22) | 3 (3.26) | |
| Unknown or not reported | 1 (2.13) | 2 (4.44) | 3 (3.26) | |
| Ethnicity | | | | 1.00 |
| Not Hispanic or Latinx | 45 (95.74) | 44 (97.78) | 89 (96.74) | |
| Hispanic or Latinx | 2 (4.26) | 1 (2.22) | 3 (3.26) | |
| Most advanced degree | | | | .080 |
| Master's Degree[b] | 37 (78.72) | 30 (66.67) | 67 (72.83) | |
| Doctoral Degree | 2 (4.26) | 1 (2.22) | 3 (3.26) | |
| Professional Degree | 6 (12.77) | 4 (8.89) | 10 (10.87) | |
| Other Degree | 2 (4.26) | 10 (22.22) | 12 (13.04) | |
| Years of overall experience | | | | .322 |
| 1 to 5 years | 15 (31.91) | 18 (40.00) | 33 (35.87) | |
| 6 to 10 years | 10 (21.28) | 7 (15.56) | 17 (18.48) | |
| 11 to 15 years | 8 (17.02) | 3 (6.67) | 11 (11.96) | |
| 16 to 19 years | 3 (6.38) | 7 (15.56) | 10 (10.87) | |
| 20 years or more | 11 (23.40) | 10 (22.22) | 21 (22.83) | |
| BASIS mechanisms | | | | |
| Attitudes, *M* (*SD*) | 3.41 (0.46) | 3.36 (0.42) | 3.38 (0.44) | .341[c] |
| Injunctive norms, *M* (*SD*) | 77.84 (13.30) | 76.35 (13.45) | 77.12 (13.47) | .480[c] |
| Descriptive norms, *M* (*SD*) | 77.60 (14.99) | 74.76 (16.05) | 76.21 (15.50) | .491[c] |
| Self-efficacy, *M* (*SD*) | 3.65 (0.35) | 3.59 (0.36) | 3.62 (0.35) | .089[c] |
| Implementation intentions, *M* (*SD*) | 6.68 (0.55) | 6.72 (0.52) | 6.70 (0.54) | .448[c] |

*Note.* BASIS = Beliefs and Attitudes for Successful Implementation in Schools; AC = Attention control. [a]*p*-values derived from chi-square for counts (i.e., demographic counts) and t-tests for BASIS mechanisms. [b]A minimum of a Master's degree was required for participation in the study. [c]Comparing baseline equivalence.

### Clinical Intervention: Trauma-Focused Cognitive Behavioral Therapy

TF-CBT is a well-established, evidence-based intervention for youth ages 3-18 who have experienced trauma and exhibit associated symptoms (e.g., behavior dysregulation, depression, self-blame).[39,44,45] It includes individual sessions for both youth and caregivers and conjoint youth-caregiver sessions, typically delivered over 12-20 weekly sessions.[44] TF-CBT has nine core treatment components–psychoeducation, parenting skills, relaxation skills, affective modulation skills, cognitive coping skills, trauma narration and processing, in-vivo mastery of trauma reminders, conjoint youth-caregiver sessions, and enhancing safety and future development trajectory (summarized by "PRACTICE" acronym).[46]

Providers in both conditions received the same TF-CBT training and consultation: a) an 8-10 hour, self-paced, online training; b) a three-day, group-based (*n* = 2-9 providers from a condition per group), live web-based training with a TF-CBT-certified trainer/consultant; and c) virtual clinical group consultation sessions (*n* = 12-19 providers from a condition per group). The TF-CBT consultation schedule was condensed to one to two sessions per month across a 6-10 month time frame to account for both providers' and TF-CBT consultants' availability across the school year.

### Study Conditions

#### *BASIS Condition*

Forty-seven providers received BASIS (Year 1: *n* = 13; Year 2: *n* = 17; Year 3: *n* = 18) pre- and post-sessions, in groups of 3-10 providers (pre-session = 3.5 hrs., post-session = 2 hrs.). BASIS was facilitated by a licensed social worker (R.B.) with extensive school-based clinical experience, formal Motivational Interviewing training, and TF-CBT certification, supported by cofacilitators (M.L.H., J.L.M., M.L., C.R.C., C.M.C.) and a technology assistant (M.J.). Sessions used structured scripts and prompts that included individual reflection, small-group problem-solving, and large-group discussion, with motivational interviewing strategies used throughout to elicit change talk, reinforce insights, and support action planning.[35,36]

Approximately 30 days post-TF-CBT training and before they received their first follow-up survey, an asynchronous online booster session link was emailed to all BASIS providers. The booster asked providers about the extent to which they had implemented, or planned to implement, TF-CBT (i.e., started PRAC components with at least one student) and, based on the response to this question, provided tailored content designed to increase implementation intentions or maintain self-efficacy. Around half of BASIS providers ($n$ = 22) spent an average of 7 minutes (range = 5-10) in the booster session.

***BASIS Fidelity.*** Fidelity was measured using an instrument developed for previous BASIS studies, operationalized as the delivery of intervention-specific informational content (e.g., the role of trauma in students' lives, importance of intervention fidelity), activities (e.g., problem-solving planning) delivered via the planned PowerPoint slide-deck, and cueing participants for engagement. BASIS delivery also included the use of motivational interviewing techniques, strategic education, and an elicit-provide-elicit framework; asking providers about their experience or knowledge, offering information aligned with supportive beliefs for behavior change, and eliciting understanding and change statements through pre-implementation planning, problem-solving planning, and action planning. For details on BASIS components and its delivery see Hugh et al., 2026 and Lyon et al., 2021. Fidelity was evaluated via video recordings by two trained, independent observers; disagreements were rare and resolved through consensus (67-93% agreement, $M$ = 86%). Fidelity was scored on a rating scale of 0-4 within segments across three dimensions adherence to content ($M$ = 3.27, $SD$ = 0.30, range = 2.73-3.47), competence of delivery ($M$ = 3.42, $SD$ = 0.43, range 2.73-3.47), and participant responsiveness ($M$ = 3.35, $SD$ = 0.34, range = 2.75-3.67), consistent with prior trials.[26,30,31,35,36]

***Attention Control Condition***

The AC condition was developed from traditional EBP training for SBMH providers[47,48] to control for dosage, structure, and facilitator effects, while using didactic content only. The pre-session (3 hrs.), covered EBP definitions and rationale, clinical outcomes associated with use of specific EBPs, and aspects of fidelity. The post-session (1.5-2 hrs.) provided opportunities to reflect on TF-CBT and its core components and discuss the outcomes associated with TF-CBT individually and in groups. The AC did not include strategic education, MI, pre-implementation planning, problem-solving planning, or action planning.

Approximately 30 days post-TF-CBT training and before providers received their first follow-up survey, an asynchronous AC booster session link was emailed to all AC providers. The AC booster prompted providers to reflect on and describe TF-CBT and identify and define its core components; 53.7% ($n$ = 22) providers spent an average of 2 minutes (range = 2-5) in the booster session.

**Measures**

Self-report measures were collected from SBMH providers via online REDCap surveys at 13 timepoints (Figure 3): after consent (T0), post-TF-CBT self-paced, online training (T1), before and after the BASIS or AC pre-session (T2-T3), before and after the BASIS or AC post-session (T4-T5), and during active implementation at 1-, 3-, 6-, and 9-months after the BASIS or AC post-session (T6-T9), and during sustainment at 12-, 15-, and 18-months after the BASIS or AC post-session (T10-T12). Of note, T0-T9 took place over the first academic year after enrolling in the study and T12 begins the second academic year.

Figure 3

*Trial Timeline with Major Milestones*

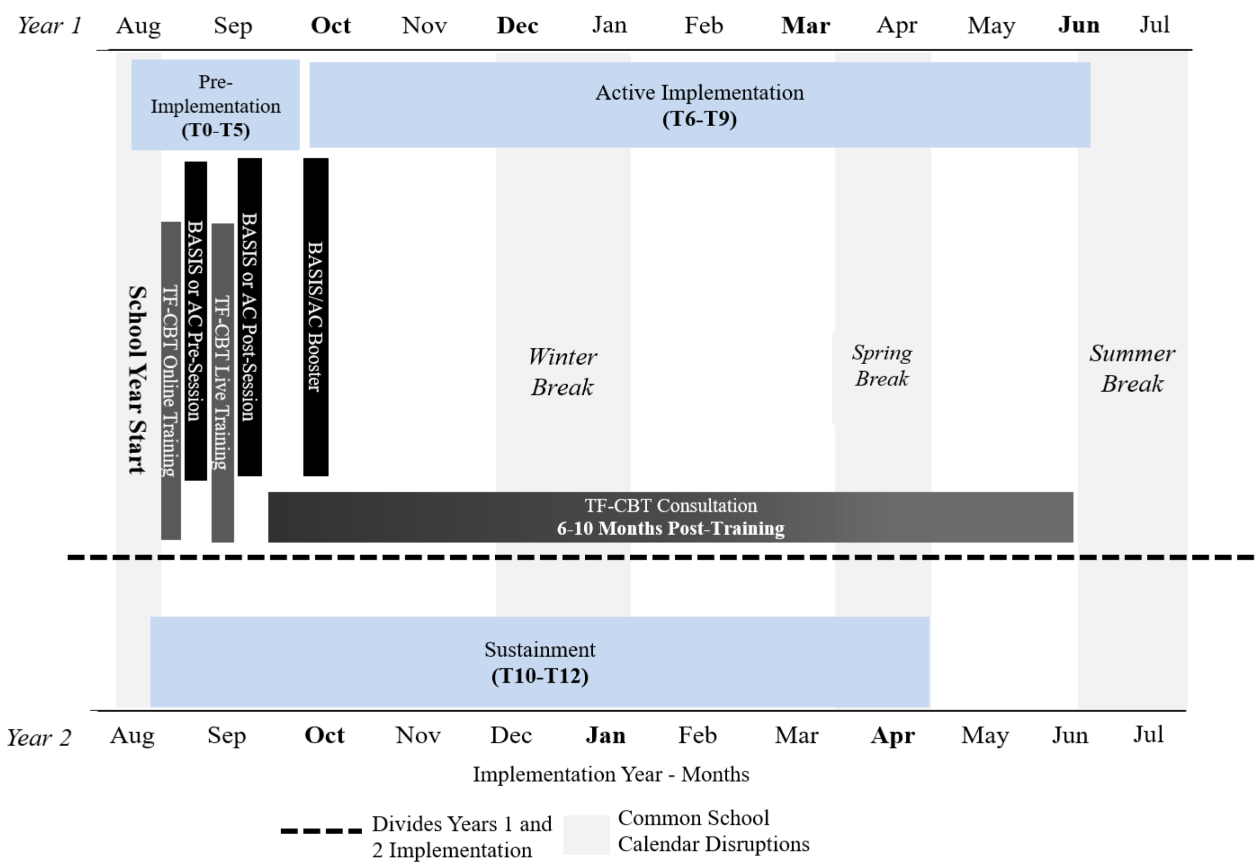

***Demographics***

Providers' demographics including age, gender, race and ethnicity, education, and years of experience were self-reported at baseline.

***SBMH Providers' Implementation Mechanisms***

**Attitudes.** The school-adapted *Evidence-Based Attitudes Scale* (EBPAS)[49,50] is a 16-item questionnaire assessing overall attitudes towards using new programs, practices, and interventions (α = .85) and four subscales: 1) appeal, 2) openness to new practices, 3) likelihood of adopting, and 4) perceived divergence between current and new practices. Providers rated their agreement with each item (e.g., *Research-based methods/interventions are not useful in practice*) on a 5-point scale ranging from 0 (not at all) to 4 (a very great extent).

**Subjective norms**. The *Modified Subjective Norms measure*[51,52] includes 8 items assessing injunctive (what a social group would approve of; 4-items, α = .75) and descriptive (how a social group actually behaves; 4-items, α = .79) norms regarding EBP delivery. Items were rated on a sliding scale from None (0) to All (100). A sample item asked *How many other people in your role believe it's an expected part of the job to use an evidence-based trauma intervention with students?*

**Self-efficacy**. The 10-item modified *Teacher Self-Efficacy Scale*[53] assesses perceived behavioral control in EBP implementation. Item wording was modified to be consistent with self-efficacy as it relates to EBP implementation (e.g., *I am confident in my ability to learn and apply effective practices to address students' social-emotional needs*). Providers rate how true each item is using a 4-point scale of 1 (Not at all true) to 4 (Very true). The scale demonstrated good internal consistency at both pre-test (α = .86) and post-test (α = .87).

**Implementation intentions.** An intentions to use measure[30,52] includes 4 items, modified to specifically assess SBMH providers' intentions to use TF-CBT (e.g., *I plan to use TF-CBT with students who have trauma*). Providers rated how much they agree with each item using a 7-point scale of 1 (strongly disagree) to 7 (strongly agree). The scale demonstrated good internal consistency at both pre-test (α = .87) and post-test (α = .90).

***TF-CBT Adoption and Completion Outcomes***

Two items were used to measure adoption. First, the item *To how many students have you given the CPSS this school year?,* was used to determine screener adoption or whether the provider had administered the CPSS-V (i.e., the *Child PTSD Symptom Scale-V*[54] is a screener of PTSD symptoms and functioning, and is the first step in assessing youth trauma and if TF-CBT implementation is needed) with at least one student. Second, the item, *Including the*

*students from [screener administration question], how many students have you started the PRAC components of TF-CBT with this school year?* was used to determine provider TF-CBT adoption. One item was used to determine TF-CBT completion: *With how many students have you completed TF-CBT (delivered all PRACTICE components)?*

**Analytic Plan**

***BASIS Effects on Implementation Mechanisms***

To answer our first research question, two sequential mixed-effects models were applied, with a unique model run for each mechanism (attitudes, norms, self-efficacy, intentions) as an outcome. Model 1 included time as the independent variable split into three piecewise variables: 1) time centered on the last day of TF-CBT training (T1-T5), 2) time centered at the end of active implementation (T6-T9, approx. 9 months), and 3) time centered at the end of sustainment (T10-T12, approx. 8 months). Time was considered a random effect in all models to adjust standard error caused by nesting timepoints within providers. Model 2 added condition (BASIS = 1, AC = 0) and an interaction term between the piecewise timepoints and condition to examine whether trajectories differed according to condition. Model fit was assessed via Akaike Information Criterion (AIC), Bayesian Information Criterion (BIC), and -2Log Likelihood (-2LL) with chi-square comparisons. Full Maximum Likelihood was used to account for missing data.[55]

***BASIS Effects on Adoption and Completion Outcomes and Pacing***

Adoption and completion outcomes were defined as three binary (yes = 1, no = 0) variables indicating: 1) screener adoption (administering the CPSS-V); 2) TF-CBT adoption (initiated a TF-CBT session); and 3) TF-CBT completion (completed the TF-CBT series (12-20 weekly sessions). Four analytic methods were used to evaluate the effects of BASIS on the three outcomes. First, we examined *the probability* of each outcome (screener adoption, TF-CBT adoption, and TF-CBT completion) occurring over the course of the study, and whether this differed by condition, from the beginning of the active implementation phase (T6-T9) to the end of sustainment (T9-T12), using time-to-event analysis and Kaplan-Meier curve.[55] Providers were considered "adopters" or "completers" the first time they endorsed any outcome (e.g., screener adoption, TF-CBT adoption, and TF-CBT adoption) with any student. Providers were considered censored if they dropped out or if they did not adopt or complete by the end of the sustainment phase. The time-to-event analysis examined whether the Kaplan-Meier curves (i.e., probability of adoption/completion) differed for BASIS, as compared to AC providers.

Next, chi-square tests compared conditions *likelihood* of each outcome at each of the three major timepoints: one month into active implementation (T6), end of active implementation (T9), and end of sustainment (T12).

Third, we assessed average differences between conditions on pacing of each outcome (i.e., first timepoint at which they reported conducting each outcome) using Mann-Whitney U Tests due to small sample size and non-normality. Listwise deletion of missing data was employed to conduct the chi-square and Mann-Whitney U tests which resulted in 80 providers.

Lastly, an exploratory analysis tested whether <u>proximal implementation mechanisms</u> immediately after BASIS/AC post-session (T5), predicted *pacing* (i.e., the first timepoint at which they reported) of each outcome (i.e., time to screener adoption, TF-CBT adoption, and TF-CBT adoption with at least one student) using a non-parametric GLM gamma log link, given skewed data distributions.

### Results

#### BASIS Effects on Implementation Mechanisms

***Attitudes***

Regardless of time, providers' overall average attitude score was 3.38 (*SD* = 0.44; $M_{\text{BASIS}}$ = 3.41, $SD_{\text{BASIS}}$ = 0.46; $M_{\text{AC}}$ = 3.36, $SD_{\text{AC}}$ = 0.42). In the time-only model, regardless of condition, providers' attitudes toward EBPs marginally increased from baseline to the end of BASIS/AC post-session (B = .02, $p$ = .053), significantly decreased during active implementation (B = -.06, $p$ = .002; Table 3 for mixed-effects models), and had no significant change during sustainment. When the BASIS condition and BASIS*time interaction were included in the model, model fit did not significantly improve (Table 2 for model fit). Neither BASIS condition nor interaction terms significantly predicted attitudes.

***Subjective Norms***

Providers' overall average injunctive and descriptive norms were 77.12 (*SD* = 13.47; $M_{\text{BASIS}}$ = 77.84, $SD_{\text{BASIS}}$ = 13.30; $M_{\text{AC}}$ = 76.35, $SD_{\text{AC}}$ = 13.45) and 76.21 (*SD* = 15.50; $M_{\text{BASIS}}$ = 77.60, $SD_{\text{BASIS}}$ = 14.99; $M_{\text{AC}}$ = 74.76, $SD_{\text{AC}}$ = 16.05), respectively. Neither providers' injunctive nor descriptive norms changed significantly over time in the time-only model. When the BASIS condition and the BASIS*time interaction were added to the model, the model fit improved for injunctive norms only. On average, BASIS providers had significantly higher injunctive (B = 3.40, $p$ = .015) and descriptive (B = 4.27, $p$ = .013) norms compared to AC providers, though norms did not differ by condition over time.

***Self-efficacy***

Providers' overall average self-efficacy score was 3.62 (*SD* = 0.35; $M_{\text{BASIS}}$ = 3.65, $SD_{\text{BASIS}}$ = 0.35; $M_{\text{AC}}$ = 3.59, $SD_{\text{AC}}$ = 0.36). In the time-only model, regardless of condition, providers' self-efficacy did not change significantly from baseline to the end of BASIS/AC post-session, though they significantly decreased during active implementation (B = -0.07, $p < .001$)

and significantly increased during sustainment (B = 0.07, $p = .002$). When the BASIS condition and the BASIS*time interaction were added to the model, there was no change in model fit; BASIS and AC providers did not demonstrate any average differences in self-efficacy, nor were there any significant differences in change over time in self-efficacy between conditions. However, similar to the time-only model, regardless of condition, self-efficacy significantly decreased during active implementation (B = -0.065, $p < .002$) and significantly increased during sustainment (B = 0.085, $p = .003$; Table 3).

***Implementation Intentions***

Providers' overall average implementation intention was 6.70 ($SD = 0.54$; $M_{\text{BASIS}} = 6.68$, $SD_{\text{BASIS}} = 0.55$; $M_{\text{AC}} = 6.72$, $SD_{\text{AC}} = 0.52$). In the time-only model, regardless of condition, providers' implementation intentions did not change significantly from baseline to the end of BASIS/AC post-session, significantly decreased during active implementation (B = -0.27, $p < .001$), and had no significant change during sustainment. When the BASIS condition and the BASIS*time interaction were added to the model there was no significant increase in model fit. Regardless of condition, there was a significant decrease during the active phase (B = -0.27, $p < .001$) and increase during the sustainment phase (B = 0.13, $p = 0.033$). There was also a significant interaction between time and condition during the sustainment phase (B = -0.33, $p < .001$), such that BASIS providers' implementation intentions decreased significantly more than AC providers.

Table 2

*Model Fit Comparisons for Implementation Mechanisms*

| **Outcome** | **Goodness of fit** | **Model 1[a]** | **Model 2[b]** | **Model 1 vs. Model 2 *p*-value[c]** |
|---|---|---|---|---|
| Attitudes | *df* | 7 | 11 | |
| | -2LL | 785.05 | 783.76 | .863 |
| | AIC | 799.05 | 805.76 | |

| Outcome | Goodness of fit | Model 1[a] | Model 2[b] | Model 1 vs. Model 2 *p*-value[c] |
|---|---|---|---|---|
| | BIC | 833.61 | 860.07 | |
| Injunctive norms | *df* | 7 | 11 | |
| | -2LL | 7959.79 | 7942.63 | .002** |
| | AIC | 7973.79 | 7964.63 | |
| | BIC | 8008.30 | 8018.86 | |
| Descriptive norms | *df* | 7 | 11 | |
| | -2LL | 7869.15 | 7872.45 | 1.00 |
| | AIC | 7883.15 | 7894.45 | |
| | BIC | 7917.67 | 7948.69 | |
| Self-efficacy | *df* | 7 | 11 | |
| | -2LL | 540.91 | 543.33 | 1.00 |
| | AIC | 554.91 | 565.33 | |
| | BIC | 589.43 | 619.58 | |
| Implementation intentions | *df* | 7 | 11 | |
| | -2LL | 2201.19 | 2199.98 | .880 |
| | AIC | 2215.93 | 2221.98 | |
| | BIC | 2249.64 | 2276.11 | |

*Note.*-2LL = -2 Log Likelihood; AIC = Akaike's Information Criterion; BIC = Schwarz's Bayesian Information Criterion.

[a]Model 1 is a multilevel model that nests time within providers, with piecewise time, and condition as predictor and specific BASIS mechanism as outcome. [b]Model 2 is a multilevel model that builds on model 1 by adding interaction with each piecewise time and condition. [c]*p-value* was calculated using ANOVA to compare the -2 Log Likelihood between the two models.

$^{*}p < .05$; $^{**}p < .01$; $^{***}p < .001$.

Table 3

*Results of Mixed-Effects Model for Impact on Implementation Mechanism Across Time*

| | | **Model 1** (Time Only) | | | **Model 2** (Time, Condition, Time*Condition) | | |
|---|---|---|---|---|---|---|---|
| **Outcome** | **Predictor** | **β (SE)** | **95% CI** | ***p*-value** | **β (SE)** | **95% CI** | ***p*-value**[a] |
| Attitudes | Intercept | 3.34 (0.02) | (3.30, 3.38) | <.001*** | 3.30 (0.03) | (3.20, 3.40) | <.001*** |
| | Training Phase (T1-T5) | 0.02 (0.01) | (0.00, 0.05) | .053 | 0.02 (0.02) | (-0.013, 0.05) | .248 |
| | Active Phase (T6-T9) | -0.06 (0.02) | (-0.09, -0.02) | .002** | -0.04 (0.03) | (-0.09, 0.01) | .119 |
| | Sustainment Phase (T10-T12) | 0.012 (0.03) | (-0.04, 0.06) | .622 | -0.016 (0.03) | (-0.075, 0.04) | .583 |
| | BASIS | - | - | - | 0.08 (0.04) | (-0.002, 0.17) | .057 |
| | Training Phase x BASIS | - | - | - | 0.009 (0.02) | (-0.036, 0.06) | .688 |
| | Active Phase x BASIS | - | - | - | -0.03 (0.03) | (-0.103, 0.03) | .314 |
| | Sustainment Phase x BASIS | - | - | - | 0.04 (0.04) | (-0.037, 0.13) | .284 |
| Injunctive Norms | Intercept | 77.01 (0.69) | (75.66, 78.36) | <.001*** | 75.3 (0.99) | (73.30, 77.20) | <.001*** |
| | Training Phase (T1-T5) | -0.097 (0.37) | (-0.83, 0.63) | .793 | -0.43 (0.49) | (-1.40, 0.53) | .378 |
| | Active Phase (T6-T9) | -0.48 (0.56) | (-1.59, 0.63) | .398 | 0.20 (0.78) | (-1.34, 1.73) | .803 |
| | Sustainment Phase (T10-T12) | 0.20 (0.79) | (-1.35, 1.74) | .801 | 0.21 (1.00) | (-1.76, 2.17) | .838 |
| | BASIS | - | - | - | 3.40 (1.40) | (0.67, 6.10) | .015* |
| | Training Phase x BASIS | - | - | - | 0.643 (0.70) | (-0.73, 2.012) | .357 |
| | Active Phase x BASIS | - | - | - | -1.16 (1.08) | (-3.29, 0.97) | .285 |
| | Sustainment Phase x BASIS | - | - | - | -0.098 (1.37) | (-2.79, 2.60) | .943 |
| Descriptive Norms | Intercept | 75.12 (0.78) | (73.58, 76.66) | <.001*** | 72.94 (1.23) | (70.52, 75.36) | <.001*** |
| | Training Phase (T1-T5) | 0.09 (0.54) | (-0.97, 1.15) | .868 | -0.26 (0.86) | (-1.96, 1.43) | .760 |
| | Active Phase (T6-T9) | -1.20 (0.76) | (-2.69, 0.29) | .115 | -0.60 (1.24) | (-3.03, 1.84) | .629 |
| | Sustainment Phase (T10-T12) | 0.46 (0.94) | (-1.40, 2.32) | .626 | -0.43 (0.85) | (-2.09, 1.24) | .614 |
| | BASIS | - | - | - | 4.27 (1.71) | (0.91, 7.64) | .013* |
| | Training Phase x BASIS | - | - | - | 0.68 (1.23) | (-1.75, 3.10) | .582 |
| | Active Phase x BASIS | - | - | - | -1.23 (1.70) | (-4.58, 2.13) | .472 |
| | Sustainment Phase x BASIS | - | - | - | 1.73 (1.16) | (-0.55, 4.00) | .137 |
| Self-efficacy | Intercept | 3.6 (0.019) | (3.5, 3.61) | <.001*** | 3.50 (0.03) | (3.49, 3.60) | <.001*** |
| | Training Phase (T1-T5) | 0.005 (0.01) | (-0.01, 0.02) | .599 | 0.003 (0.01) | (-0.02, 0.03) | .788 |
| | Active Phase (T6-T9) | -0.07 (0.02) | (-0.10, -0.04) | <.001*** | -0.065 (0.02) | (-0.11, -0.02) | .002** |
| | Sustainment Phase (T10-T12) | 0.07 (0.02) | (0.024, 0.11) | .002** | 0.085 (0.03) | (0.03, 0.14) | .003** |
| | BASIS | - | - | - | 0.065 (0.04) | (-0.01, 0.14) | .089 |
| | Training Phase x BASIS | - | - | - | 0.003 (0.02) | (-0.03, 0.04) | .886 |
| | Active Phase x BASIS | - | - | - | -0.013 (0.03) | (-0.07, 0.05) | .663 |
| | Sustainment Phase x BASIS | - | - | - | -0.04 (0.04) | (-0.12. 0.04) | .296 |

| Outcome | Predictor | Model 1 (Time Only) β (SE) | Model 1 95% CI | Model 1 *p*-value | Model 2 (Time, Condition, Time*Condition) β (SE) | Model 2 95% CI | Model 2 *p*-value[a] |
|---|---|---|---|---|---|---|---|
| Implementation intentions | Intercept | 6.61 (0.04) | (6.53, 6.70) | <.001*** | 6.60 (0.06) | (6.47, 6.71) | <.001*** |
| | Training Phase (T1-T5) | 0.015 (0.02) | (-0.03, 0.06) | .501 | 0.01 (0.03) | (-0.05, 0.07) | .743 |
| | Active Phase (T6-T9) | -0.27 (0.04) | (-0.34, -0.19) | <.001*** | -0.27 (0.05) | (-0.37, -0.16) | <.001*** |
| | Sustainment Phase (T10-T12) | -0.04 (0.05) | (-0.14, 0.06) | .428 | 0.13 (0.06) | (0.01, 0.26) | .033* |
| | BASIS | - | - | - | 0.046 (0.09) | (-0.13, 0.22) | .602 |
| | Training Phase x BASIS | - | - | - | 0.01 (0.04) | (-0.08, 0.096) | .812 |
| | Active Phase x BASIS | - | - | - | 0.013 (0.07) | (-0.13, 0.16) | .858 |
| | Sustainment Phase x BASIS | - | - | - | -0.33 (0.09) | (-0.49, -0.16) | <.001*** |

*Note*. $^{*}p < .05$; $^{**}p < .01$; $^{***}p < .001$. [a]*p*-value for the estimates of fixed effects derived from *t*-test.

### BASIS Effects on Adoption and Completion Outcomes and Pacing

#### *Screener Adoption*

Of 80 providers (40 per condition), 34 BASIS (85%) and 26 AC (65%) providers administered the screener with at least one student (Figure 4). Time-to-event analysis (Table 4) demonstrated that over the course of the study, BASIS providers were not significantly more likely to adopt the screener with at least one student compared to AC providers. Chi-square tests (Table 5) indicated no significant difference in the number of providers who adopted the screener between BASIS (15%) and AC (20%) one month after BASIS/AC post-session (T6). However, by the end of active implementation, BASIS providers were on average more likely to have ever adopted at least one screener ($n_{\text{BASIS}}$ = 34, 85%; $n_{\text{AC}}$ = 25, 62.5%; $p$ = .022), which maintained through sustainment (T12), (85% vs. 65%, respectively; $p$ = .039). Furthermore, on average, there was no significant difference in who first adopted a screener between conditions (Table 6 for pace results). Both BASIS and AC providers adopted their first screener between 3 and 6 months into active implementation (T7-T8).

Figure 4

*Rate of Screener Adoption by Condition by Timepoint*

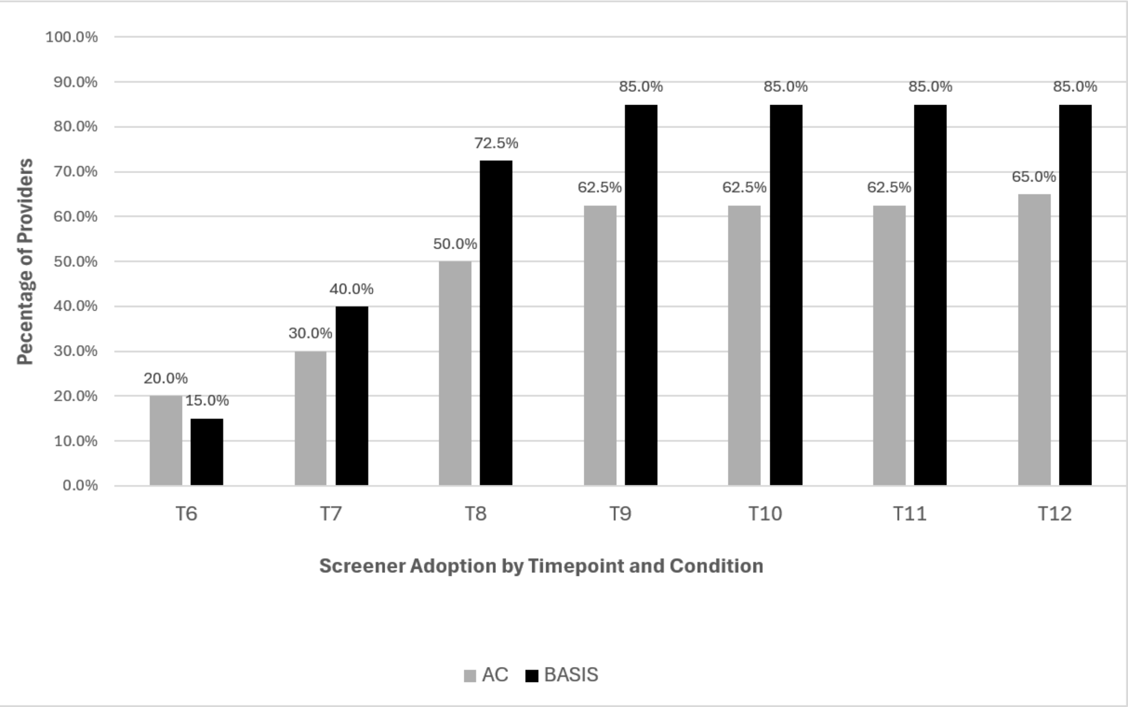

#### *TF-CBT Initiation*

Among providers who adopted the screener, 32 BASIS (94.12%) and all AC providers adopted TF-CBT with at least one student (Figure 5). The time-to-event curves suggested that BASIS providers had a marginally higher likelihood of adopting TF-CBT with at least one student over the course of the full study period ($n_{BASIS}$ = 32, 80%; $n_{AC}$ = 26, 65%; $p$ = .065). Chi-square results indicated that there was no significant difference in the number of providers who adopted TF-CBT with at least one student one month after BASIS/AC post-session (T6). However, at the end of active implementation (T9), there was a marginal difference in TF-CBT adoption with at least one student ($p$ = .051); more BASIS providers ($n_{BASIS}$ = 32; 80%) reported adopting TF-CBT with at least one student compared to AC providers ($n_{AC}$ = 24; 60%). By the end of sustainment (T12), there was no significant difference between the number of BASIS and AC providers who adopted TF-CBT. On average, BASIS providers adopted TF-CBT marginally earlier ($p$ = .067; Table 6). Specifically, BASIS providers adopted between 3 and 6 months into active implementation (T7-T8; $M_{\text{BASIS}} = 7.66$, $SD_{\text{BASIS}} = 0.75$), compared to AC providers who adopted TF-CBT after 6 months (T8; $M_{\text{AC}} = 8.04$, $SD_{\text{AC}} = 0.92$).

Figure 5

*Rate of TF-CBT Adoption by Condition by Timepoint*

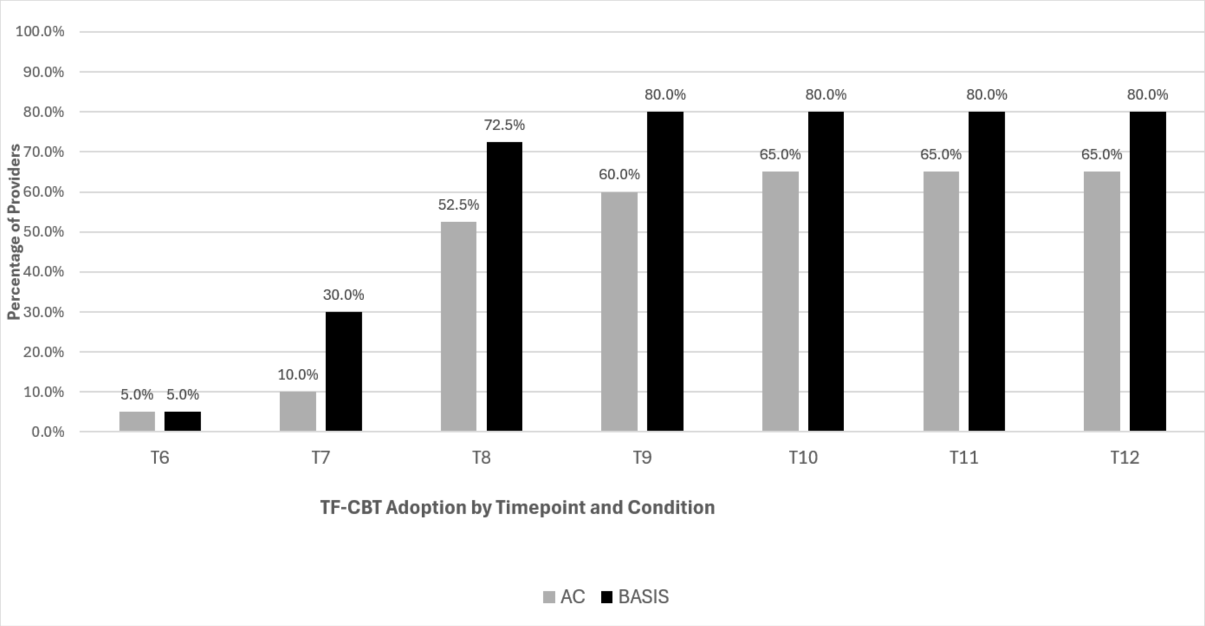

#### ***TF-CBT Completion***

Among providers who adopted TF-CBT, 11 BASIS (34.38%) and 12 AC (46.15%) providers completed TF-CBT with at least one student (Figure 6). There were no significant differences between the time-to-event curves of BASIS ($n_{\text{BASIS}} = 11$; 27.5%) and AC ($n_{\text{AC}} = 12$; 30%) providers' TF-CBT completion. Chi-square results indicated that there was no significant difference in the number of providers who completed TF-CBT with at least one student at the end of active implementation (T9) or sustainment (T12). Furthermore, on average, there was no significant difference in who completed TF-CBT first between conditions ($p = 0.235$; Table 6). Specifically, BASIS providers first completed TF-CBT between the end of active implementation and beginning of sustainment (T9-T10; $M_{\text{BASIS}} = 9.91$, $SD_{\text{BASIS}} = 1.76$), while

AC providers first completed it by the end of active implementation (T9; $M_{AC} = 9.00$, $SD_{AC} = 0.74$).

Figure 6

*Rate of TF-CBT Completion by Condition by Timepoint*

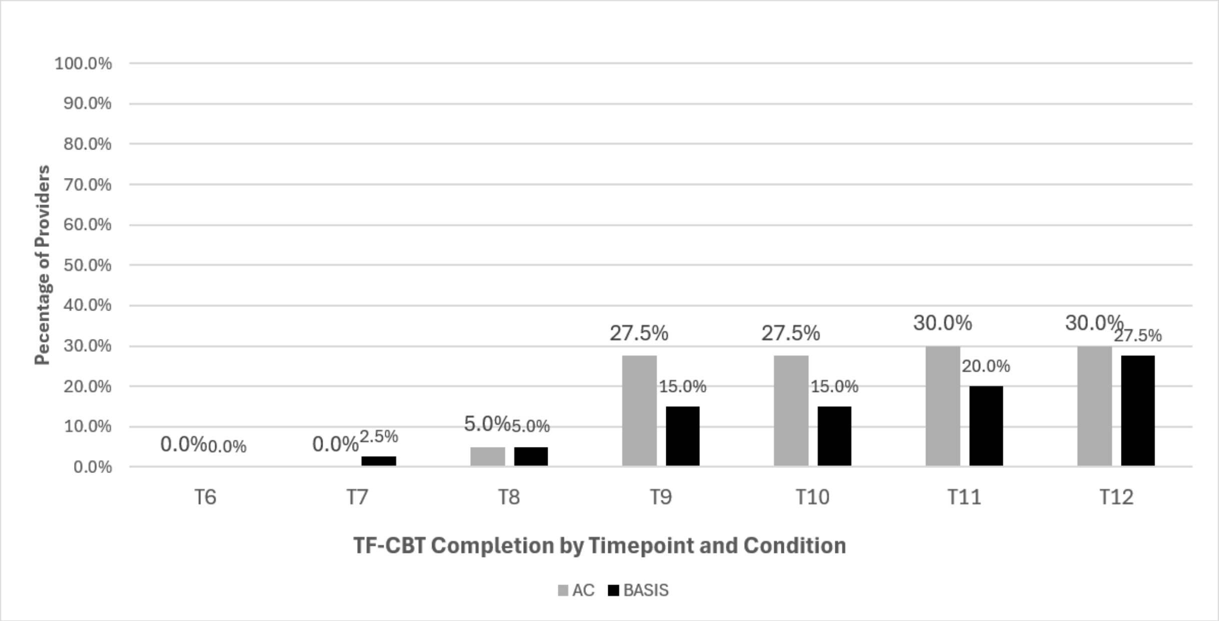

Table 4

*Time-to-Event Analysis of Adoption and Completion Outcomes by Condition*

| **Outcome** | **Condition** | ***N*** | **Events (*n*)** | **Censored *n* (%)** | **Mean time to event[a] (95% CI)** | **Median time to event (timepoint) (95% CI)** | ***p*-value[b]** |
|---|---|---|---|---|---|---|---|
| Time to first screener adoption | AC | 40 | 26 | 14 (35.00) | 8.62 (7.89, 9.35) | 8 (7.35, 8.65) | .136 |
| | BASIS | 40 | 34 | 6 (15.00) | 7.91 (7.42, 8.40) | 8 (7.63, 8.37) | |
| Time to first TF-CBT adoption | AC | 40 | 26 | 14 (35.00) | 8.87 (8.28, 9.46) | 8 (7.69, 8.32) | .065 |
| | BASIS | 40 | 32 | 8 (20.00) | 8.23 (8.14, 8.94) | 8 (7.71, 8.29) | |
| Time to first TF-CBT completion | AC | 40 | 12 | 28 (70.00) | 10.85 (10.32, 11.38) | - | .485 |
| | BASIS | 40 | 11 | 29 (72.50) | 11.36 (10.91, 11.81) | - | |

*Note.* [a]Mean to event is the average timepoint the event occurs in. [b]*p*-value derived from log-rank (Mantel-Cox) test comparing AC and BASIS conditions

Table 5

*Results of the Chi-Square Test*

| **Outcome** | **Condition** | ***N*** | **Yes** ***n*** **(%)** | **No** ***n*** **(%)** | ***p*-value**[a] |
|---|---|---|---|---|---|
| **Screener Adoption** | | | | | |
| By one month after BASIS/AC post-session with at least one student | AC | 40 | 8 (20.00) | 32 (80.00) | .560 |
| | BASIS | 40 | 6 (15.00) | 34 (85.00) | |
| | Total[c] | 80 | 14 (17.50) | 66 (82.50) | |
| By the end of active implementation phase with at least one student | AC | 40 | 25 (62.50) | 15 (37.50) | .022* |
| | BASIS | 40 | 34 (85.00) | 6 (15.00) | |
| | Total[c] | 80 | 59 (73.75) | 21 (26.25) | |
| By end of sustainment phase with at least one student | AC | 40 | 26 (65.00) | 14 (35.00) | .039* |
| | BASIS | 40 | 34 (85.00) | 6 (15.00) | |
| | Total[c] | 80 | 60 (75.00) | 20 (25.00) | |
| **TF-CBT Adoption** | | | | | |
| By one month after BASIS/AC post-session with at least one student | AC | 40 | 2 (5.00) | 38 (95.00) | 1.00 |
| | BASIS | 40 | 2 (5.00) | 38 (95.00) | |
| | Total[c] | 80 | 4 (5.00) | 76 (95.00) | |
| By the end of active implementation phase with at least one student | AC | 40 | 24 (60.00) | 16 (40.00) | .051 |
| | BASIS | 40 | 32 (80.00) | 8 (20.00) | |
| | Total[c] | 80 | 56 (70.00) | 24 (30.00) | |
| By end of sustainment phase with at least one student | AC | 40 | 26 (65.00) | 14 (35.00) | .133 |
| | BASIS | 40 | 32 (80.00) | 8 (20.00) | |
| | Total[c] | 80 | 58 (72.50) | 22 (27.50) | |
| **TF-CBT Completion**[b] | | | | | |
| By the end of active implementation phase with at least one student | AC | 40 | 11 (27.50) | 29 (72.50) | .172 |
| | BASIS | 40 | 6 (15.00) | 34 (85.00) | |
| | Total[c] | 80 | 17 (21.25) | 63 (78.75) | |
| By end of sustainment phase with at least one student | AC | 40 | 12 (30.00) | 28 (70.0) | .805 |
| | BASIS | 40 | 11 (27.50) | 29 (72.50) | |
| | Total[c] | 80 | 23 (28.75) | 57 (71.25) | |

*Note.* $^{*}p < .05$

[a]*p*-value derived from Pearson's chi-square test comparing AC and BASIS conditions. [b]Given completion by one month after BASIS/AC post-session was not attenable due to the length of time needed to complete TF-CBT, this was removed from the table ($n = 0$).

[c]Listwise deleted missing values, hence the total *n* is 80 instead of 92.

Table 6

*Mann-Whitney U Test Comparing Pacing of Adoption and Completion Outcomes by Condition*

| **Outcome** | **Condition** | ***n*** | **Time of first implementation outcome *M* (*SD*)** | **Mean rank** | **U statistic** | ***p*-value[a]** |
|---|---|---|---|---|---|---|
| Time to first screener adoption with at least one student | AC | 26 | 7.50 (0.96) | 30.40 | 790.50 | .969 |
| | BASIS | 34 | 7.58 (1.45) | 30.57 | 1039.50 | |
| Time to first TF-CBT adoption with at least one student | AC | 26 | 8.04 (0.92) | 33.50 | 871.00 | .067 |
| | BASIS | 32 | 7.66 (0.75) | 26.35 | 840.00 | |
| Time to first TF-CBT completion with at least one student | AC | 12 | 9.00 (0.74) | 10.33 | 124.00 | .235 |
| | BASIS | 11 | 9.91 (1.76) | 13.82 | 152.00 | |

*Note.* [a]*p*-value derived from Mann-Whitney U Test comparing AC and BASIS conditions

***Implementation Mechanism on Pacing of Adoption and Completion Outcomes***

After listwise deletion, there were 58 BASIS and 23 AC providers included in the regression analyses (Table 7). Controlling for condition, only attitudes ($p$ = .035) and self-efficacy ($p$ = .041) measured immediately post-TF-CBT training, significantly predicted time to first screener adoption. For every unit increase in EBPAS score, there was a 10% increase in time to adopt the screener, such that providers with more positive attitudes adopted the screener later. Similarly, for every unit increase in self-efficacy, there was a 13% increase in time to adopt the screener; providers with higher self-efficacy scores adopted the screener later. Neither attitudes nor self-efficacy predicted time to TF-CBT adoption or completion; further, norms and intentions did not predict any adoption or completion outcomes.

Table 7

*Implementation Mechanisms Predicting Pacing of Adoption and Completion Outcomes*

| Outcome | Predictor | *n* | β (SE) | 95% CI | Exp (β) | *p*-value[a] |
|---|---|---|---|---|---|---|
| Time to first screener adoption | Attitudes | 60 | 0.09 (0.04) | (0.01, 0.18) | 1.10 | .035* |
| | Injunctive norms | 60 | 0.002 (0.001) | (-0.001, 0.005) | 1.00 | .171 |
| | Descriptive norms | 60 | 0.001 (0.001) | (-0.001, 0.003) | 1.00 | .496 |
| | Self-efficacy | 60 | 0.12 (0.06) | (0.01, 0.24) | 1.13 | .041* |
| | Implementation intentions | 59 | 0.05 (0.07) | (-0.08, 0.19) | 1.06 | .423 |
| Time to first TF-CBT adoption | Attitudes | 58 | 0.04 (0.03) | (-0.02, 0.10) | 1.04 | .236 |
| | Injunctive norms | 58 | 0.001 (0.001) | (-0.001, 0.003) | 1.00 | .252 |
| | Descriptive norms | 58 | 0 (0.008) | (-0.002, 0.002) | 1.00 | .996 |
| | Self-efficacy | 58 | 0.061 (0.04) | (-0.02, 0.15) | 1.06 | .155 |
| | Implementation intentions | 57 | 0.07 (0.04) | (-0.01, 0.16) | 1.08 | .083 |
| Time to first TF-CBT completion | Attitudes | 23 | 0.01 (0.06) | (-0.11, 0.12) | 1.01 | .926 |
| | Injunctive norms | 23 | 0.001 (0.002) | (-0.003, 0.005) | 1.00 | .504 |
| | Descriptive norms | 23 | 0.001 (0.002) | (-0.003, 0.005) | 1.00 | .584 |
| | Self-efficacy | 23 | 0.05 (0.10) | (-0.14, 0.25) | 1.05 | .603 |
| | Implementation intentions | 23 | 0.17 (0.14) | (-0.11, 0.45) | 1.19 | .228 |

*Note.* $^{*}p < .05$. [a]*p-value* for individual parameter estimate derived from Wald Chi-square test.

### Discussion

To build a more precise understanding of how implementation strategies affect adoption and completion, researchers must shift focus to include *when* changes occur.[18,56] Provider-level strategies that enable timely and continued EBP adoption and completion are needed to bring trauma-focused EBPs, like TF-CBT, into care settings quicker.[8,26,30,31] To advance knowledge of the pace of school-based implementation, this hybrid trial evaluated BASIS, a pragmatic, theoretically grounded pre-implementation strategy targeting SBMH providers' individual-level mechanisms, to assess not just whether providers adopted and completed TF-CBT but when. Our findings suggest that the timing of mechanism change matters, reflecting both the real-world constraints of delivering a complex EBP within a school calendar and the potential for specific mechanisms to differentially influence adoption and completion at different timepoints. Regardless of condition, higher self-efficacy and attitudes immediately post-TF-CBT training predicted a slower start to screener adoption. BASIS providers' implementation intentions also weakened over time. Nevertheless, BASIS increased the likelihood of screener adoption and marginally accelerated TF-CBT adoption compared to AC, though it was insufficient to support completion. These results suggest that ongoing, appropriately timed, mechanism-targeted support may be facilitative for timely and continued progression through an implementation timeline (i.e., screener adoption, TF-CBT adoption), and that pre-existing strategies may not yet be meeting this need.

#### BASIS Effects Along TF-CBT's Implementation Timeline

Our findings suggest that BASIS can support providers' pacing of adoption and completion, especially within TF-CBT's lengthy implementation window within the context of a constrained school year among other barriers. BASIS increased the likelihood that providers ever administered a screener and showed some indication of speeding TF-CBT adoption, but these advantages did not extend to completion. TF-CBT differs from many EBPs typically implemented by SBMH providers in that it was developed for clinical settings, is relatively lengthy, emphasizes caregiver involvement, and is typically delivered by only one or two providers per building.[57,58] Future studies may explore differential effects by SBMH role or training-level as some providers may feel more confident than others delivering a complex EBP based on their prior experiences. These characteristics make progression through TF-CBT's implementation timeline demanding, and may explain why BASIS effects were concentrated early. These findings, taken into context, also underscore why individual-level mechanisms may be critical targets before implementation even begins.

Beyond TF-CBT's characteristics, structural features of the school calendar such as required start and end dates, pre-scheduled breaks, and instructional times impose additional barriers.[11] Starting delivery with a student as early in the year as possible is critical to completing a full TF-CBT series. Yet we found providers in both conditions typically did not first screen or initiate TF-CBT with a student until between 3-6 months (T7-T8) into the active implementation phase. With adopting the screener and TF-CBT adoption concentrated in a narrow mid-year window, providers who delayed faced limited time to progress to later phases. Given TF-CBT requires 12-20 weekly sessions, a student screened mid-year has only a few months before the academic calendar may end delivery.

Despite barriers that may constrain progression, TF-CBT completion was observed into the sustainment phase. While no significant between-condition differences emerged, possibly due to small sample size, descriptively, the number of BASIS providers who completed TF-CBT nearly doubled from the end of active implementation to the end of the sustainment phase. These findings warrant attention as sustainability matters not only for continuity of care, but for understanding adequate pacing of adoption, as students who need TF-CBT in subsequent years are equally deserving of timely access. Together, these findings add to literature suggesting timing of implementation strategies is key,[59] and imply that strategies, like BASIS, may need to be aligned with key action windows across the implementation timeline, with supports timed to help sustain momentum and facilitate progression.

**Attitudes and Self-Efficacy Slow Screener Adoption**

Counter to expectations, providers with more positive attitudes and higher self-efficacy took longer to screen their first student, regardless of condition. Further, implementation mechanisms did not predict TF-CBT adoption or completion. While attitudes and self-efficacy may remain key mechanisms to implementation, they alone may be insufficient for adopting a complex EBP like TF-CBT. Providers may have experienced a "know-do gap," in which positive beliefs about TF-CBT were not paired with providers' "knowing" enough to "do" the intervention.[60] This suggests that more intensive, beliefs-focused support–delivered at the right point in the school year–may be needed to help providers translate positive attitudes and beliefs into timely adopters and completers. Moreover, better understanding implementation speed metrics and the mechanisms that influence them can clarify how long implementation takes and guide the identification and tailoring of strategies to increase it.

**Mechanism Deterioration Calls for Appropriately Timed Implementation Strategies**

Mechanisms were not static precursors to behavior; on average, they shifted across the school year in both conditions. BASIS providers had higher subjective norms, particularly injunctive norms. Providers' intentions, which are most predictive of implementation within actionable windows,[61] declined as opportunities to adopt and complete TF-CBT narrowed toward the end of the academic year, then rebounded in the sustainment phase as a new academic calendar began. This points to the importance of measuring both multiple implementation outcomes and mechanisms over time,[22,62] enabling future research to identify conditions under which pacing of adoption and completion, as well as other implementation outcomes, accelerates or slows.

These results highlight the need to optimize implementation strategies based on mechanisms and their timing. For instance, BASIS, as delivered (i.e., pre- and post-sessions bookending TF-CBT training, with a booster ~30 days after TF-CBT training), may have been insufficient to sustain providers across the full implementation timeline. Different mechanisms may operate differently across implementation phases, with intentions facilitating initial uptake, and norms playing a longer-term role. Subjective norms remained a stable, though non-significant, difference between conditions. Norms may have been more susceptible to fluctuating across the study given providers are periodically distanced from their professional networks (e.g., summer break), which fell outside the bounds of our timepoints and warrants further investigation. These dynamics were potentially further compounded by the COVID-19 pandemic and other natural disasters (e.g., tornados), which disrupted the school environment and separated providers from students through school virtualization. The tenuous nature of navigating one's own social expectations and pressure amidst increasingly stressful and potentially traumatizing events, may have overridden the process of subjective norms on behavior. Future research should examine whether mechanism trajectories are predictable enough to anticipate declines across implementation timelines and proactively time strategies to interrupt deterioration.[63] Beyond timing, our recruitment strategy yielded a sample spanning across all US regions, which strengthens the generalizability of our findings, but also introduces heterogeneity worth noting. Schools and districts across regions vary considerably in their political contexts surrounding student mental health needs,[64–66] with some prioritizing trauma-informed practice and mental health infrastructure more than others. Such variability may have contributed to unmeasured differences in TF-CBT adoption and completion. Future work should examine policy-level moderators, among other regional variables, to better understand how context shapes implementation.

**Limitations**

This study should be considered in light of several limitations. First, it was initiated during the first year of the COVID-19 pandemic, which significantly disrupted school structures and policies. This led to virtual TF-CBT and BASIS training and delivery, during which we occasionally experienced technical challenges. Second, providers began the trial with high scores of most theorized mechanisms (e.g., 6.70 self-efficacy) thus leaving little room for improvements. This suggests that BASIS was evaluated among providers who did not have strong deficiencies in mechanisms, and may explain the lack of effects. Additionally, mechanism measures (e.g., attitudes, norms, self-efficacy) assessed beliefs about evidence-based practices generally rather than TF-CBT which was the use case in this trial. Future highly-powered trials may examine whether BASIS effects are moderated by baseline scores; this would help clarify whether strategies are suited for providers with room to improve mechanisms. Third,

TF-CBT completion was only measured through the end of the study period, so providers still delivering TF-CBT were coded as "non-completers" despite potentially being on track to complete. Fourth, while we explored findings of marginal significance to inform future BASIS trials and opportunities to examine pacing of adoption and completion, these, along with our pace analyses, should be interpreted with caution given the small sample size and non-normality of data, which necessitated non-parametric tests. Additionally, to address our research questions/hypotheses and outcomes, we ultimately ran a large number of significance tests, which can increase the likelihood of Type 1 error. Fifth, some nuance was lost with the binary coding of adoption and completion outcomes, which while conceptually consistent with operationalizations of adoption in the literature,[22,67] does not capture variability in extent or quality and may obscure trajectories of adoption/completion. An important next step for future BASIS trials will be to collect session-level fidelity data to better understand the nuances of adoption and uptake over time.

**Conclusion**

The unique needs of EBP recipients do not wait for providers to get EBPs into practice, requiring the field to move beyond whether adoption and completion occur to also consider when. This is especially important in contexts like schools, where timing constraints shape implementation pace. Findings from this study demonstrate that implementation strategies targeting individual-level mechanisms can facilitate early EBP initiation, with implications for both future research and for students with the highest needs, who deserve equitable and timely access to care. Further investigation is needed to understand how such implementation strategies may be optimized to accelerate school-based implementation of TF-CBT and other EBPs.

**List of Abbreviations**

-2LL: -2Log Likelihood

AC: Attention Control

AIC: Akaike Information Criterion

BASIS: Beliefs and Attitudes for Successful Implementation in Schools

BIC: Bayesian Information Criterion

CPSS-V: Child PTSD Symptom Scale-V

EBP: evidence-based practice

EBPAS: Evidence-Based Attitudes Scale

K-12: kindergarten through twelfth grades

SBMH: school-based mental health

TF-CBT: Trauma-Focused Cognitive Behavior Therapy

US: United States

## Supplementary Files

This is a list of supplementary files associated with this preprint. Click to download.

- SupplementaryMaterial1.docx
